# Phylogeographic analysis reveals high genetic structure with uniform phenotypes in the paper wasp *Protonectarina sylveirae* (Hymenoptera: Vespidae)

**DOI:** 10.1371/journal.pone.0194424

**Published:** 2018-03-14

**Authors:** Marjorie da Silva, Fernando Barbosa Noll, Adriana C. Morales-Corrêa e Castro

**Affiliations:** 1 Departamento de Zoologia e Botânica, Instituto de Biociências, Letras e Ciências Exatas, Universidade Estadual Paulista “Júlio de Mesquita Filho”, São José do Rio Preto, São Paulo, Brazil; 2 Departamento de Biologia Aplicada à Agropecuária, Faculdade de Ciências Agrárias e Veterinárias, Universidade Estadual Paulista “Júlio de Mesquita Filho”, Jaboticabal, São Paulo, Brazil; National Cheng Kung University, TAIWAN

## Abstract

Swarm-founding wasps are endemic and common representatives of neotropical fauna and compose an interesting social tribe of vespids, presenting both complex social characteristics and uncommon traits for a eusocial group, such as the absence of castes with distinct morphology. The paper wasp *Protonectarina sylveirae* (Saussure) presents a broad distribution from Brazil, Argentina and Paraguay, occurring widespread in the Atlantic rainforest and arboreal Caatinga, being absent in the Amazon region. Given the peculiar distribution among swarm-founding wasps, an integrative approach to reconstruct the evolutionary history of *P*. *sylveirae* in a spatial-temporal framework was performed to investigate: the presence of genetic structure and its relationship with the geography, the evolution of distinct morphologic lineages and the possible historical event(s) in Neotropical region, which could explain the observed phylogeographic pattern. Individuals of *P*. *sylveirae* were obtained from populations of 16 areas throughout its distribution for DNA extraction and amplification of mitochondrial genes 12S, 16S and COI. Analysis of genetic diversity, construction of haplotype net, analysis of population structure and dating analysis of divergence time were performed. A morphometric analysis was also performed using 8 measures of the body of the adult (workers) to test if there are morphological distinction among populations. Thirty-five haplotypes were identified, most of them exclusively of a group and a high population structure was found. The possibility of genetic divergence because of isolation by distance was rejected. Morphological analysis pointed to a great uniformity in phenotypes, with only a small degree of differentiation between populations of south and the remaining. Divergence time analysis showed a Middle/Late Miocene origin, a period where an extensive marine ingression occurred in South America. Divergence of haplogroups began from the Plio/Pleistocene boundary and the last glacial maximum most likely modeled the current distribution of species, even though it was not the cause of genetic breaks.

## Introduction

Comprehension of the evolutionary processes that generate and maintain the biological diversity of tropical fauna has been challenging biologists for centuries and should be the underpinning of conservation strategies [1]. Current distributions result from the interaction between environmental requirements of species and geographical variation of environmental features [2]. Investigating the evolutionary history of species can increase understanding of the interactions between past climatic events and the evolutionary processes that contributed to current patterns of diversity [3–5].

Phylogeography focuses on the processes governing the distribution of genealogical lineages within species across the geographical landscape [6, 7]. Phylogeographic approaches facilitate an increased understanding of the role that historical events play in the geographical patterns of genetic variability within and among species [5, 8, 9]. One of the strengths of this discipline is the approach of micro evolutionary processes (within populations) and macro evolutionary patterns at larger spatial and temporal scales, in an integrated way, by linking heredity (processes at the level of individual pedigrees), divergence at the population level, and phylogenetic relationships among species [6, 10]. Demographic processes—drift, expansion and changes in effective population size—are consequence of biotic and abiotic conditions which a species was submitted during its evolutionary history and are reflected in genetic structure of neutral genes. The variable environmental conditions also act selecting phenotypes, once the morphological features affect the performance of individuals, impacting processes such as dispersal, colonization, and persistence [10]. Moreover, phenotypes provide information on local selective pressures and indicate a gene flow rupture history of the various populations due to a partial or total isolation between them [10, 11]. Thus, analysis of phenotypic variation within a phylogeographic approach can promote great advances in understanding how genetic and morphological divergence arises and how they behave in the face of ecological / evolutionary changes [10].

A single model of vicariance or recent climate changes cannot explainthe origin of Neotropical biota [12]. This region, which includes the south of Mexico, Central and South Americas and the Caribbean islands, is known for remarkable biodiversity [13] and is perhaps the richest terrestrial biogeographical region in terms of species [14]. Understanding the spatial patterns integrated with the molecular diversity, phenotypic variation, reproductive isolation and history of areas can provide information about speciation, clarify the historical biogeography and allow hypothesis regarding the diversification mechanisms. Thereafter, incorporating such aspects can contribute significantly to the development of strategies for conservation of ecosystems [1, 15].

Swarm-founding wasps of the Epiponini compose an interesting social tribe of vespids, presenting both complex social characteristics (intricate nest building, polygyny and swarm reproduction) and uncommon traits for a eusocial group, such as castes with the same or with a very similar morphology [16, 17]. As the name suggests, these wasps initiate their colonies by swarms, with one or more queens accompanied by a group of workers [17, 18]. As a social insect, the effective population size of *P*. *sylveirae* is very low, once there is only one or a few reproductives in the colony. The swarm strategy plus the polygyny (presence of more than one queen) greatly reduced the risk of loss of the reproductives, increasing the survive of the colony [19, 20, 21]. In these colonies, females are philopatric and the dispersion is male-biased, mostly in polygynous populations [22, 23].

**Fig 1:**
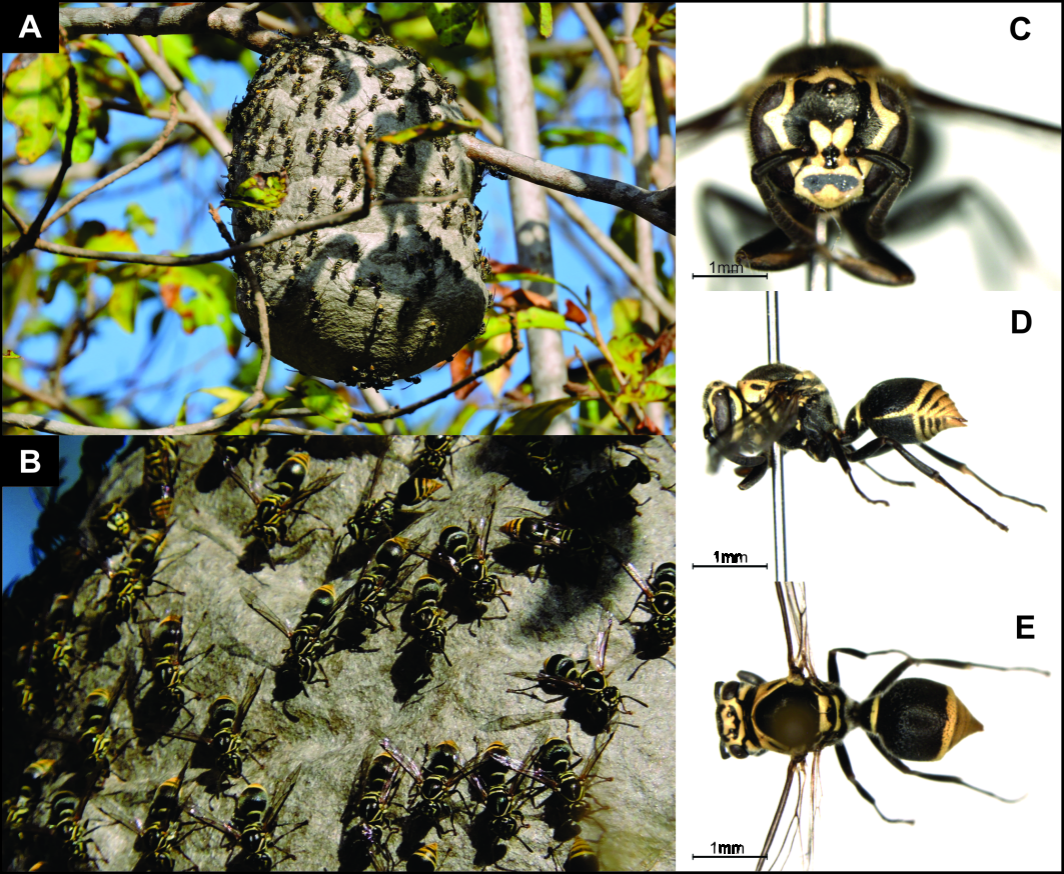
Nest and specimen of *Protonectarina sylveirae*. A. Nest in a tree from Mossoró, RN, Brazil. B. Detail of the same nest showing many wasps outside, a common behavior of this species. Frontal (C), lateral (D) and dorsal (E) views of a worker.

**Fig 2:**
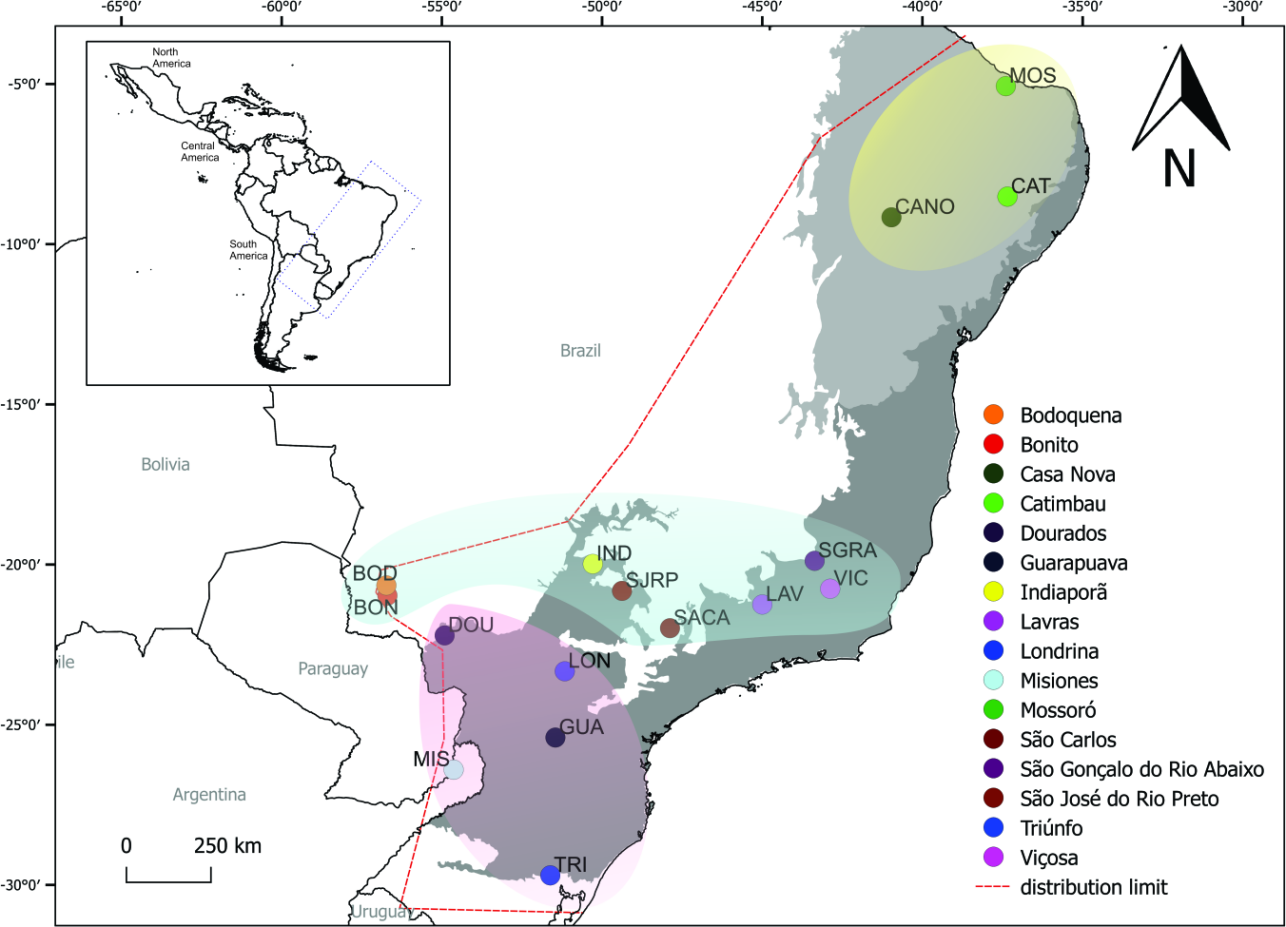
Area of occurrence of *Protonectarina sylveirae*. Map of South America showing the distribution of *P*. *silveirae* and sample locations used for analysis. Circles represent the points sampled, and the dashed line indicates the limit of species distribution according to literature data and collection records. Acronyms and colors will be matched for the haplotype network and chronogram. Areas of Atlantic Forest (dark gray) and Caatinga (light gray) are represented, as are the geographic groups of Northeast (yellow), Central (blue) and South (pink). The map was generated using the software QGIS v2.18 (http://www.qgis.org/en/site/forusers/download.html).

Common representatives of Neotropical fauna, 19 genera and approximately 234 described species compose the Epiponini tribe [17]. *Protonectarina sylveirae* (Saussure) is the only species of the genus *Protonectarina* Ducke, and these aggressive wasps form colonies of large populations that build very large and perennial arboreal nests, usually suspended from a twig [16, 24, 25]. The colonies store a considerable amount of nectar, an unusual feature among wasps [16, 24, 25] (Fig 1). The species has a broad distribution from Brazil to Argentina, specifically along the central (Minas Gerais, Mato Grosso do Sul and Goiás) and eastern regions of Brazil (Ceará, Bahia, Espírito Santo, Rio de Janeiro, São Paulo, Paraná, Santa Catarina and Rio Grande do Sul), the eastern end of Argentina and eastern Paraguay [16, 26] (Fig 2). Among the genera of Epiponini, *Protonectarina* is the only one that is not found in the Amazon region, occurring widespread in the Atlantic rainforest and arboreal Caatinga (a type of desert vegetation found exclusively in the interior northeastern of Brazil composed of a mosaic of spiny shrubs and seasonally dry forests [27].

The Brazilian Atlantic Forest is the second largest tropical forest in South America, covering an area of > 1,000,000 km^2^ along the Brazilian coast, eastern Paraguay and northeastern Argentina [28–30]. Complex factors including strong seasonality, sharp environmental gradients and orographically driven rainfall result in a diverse landscape in this ecoregion [28, 31], which includes coastal Atlantic rainforests, semi-deciduous forests, subtropical *Araucaria* (Jussieu) forests and *brejo* forests [32]. For this biome, much of the research identifies recurrent phylogeographical discontinuities for different groups of organisms; for example, bees [33], amphibians [34, 35], reptiles [36, 37], birds [38,39], bats [40] and plants [41]. Moreover, some authors infer historical connections between Atlantic rainforest and Amazonian rainforest [12, 42].

The most well-known hypothesis on the origin of diversity in the Brazilian Atlantic rainforest is based on the classical Pleistocene Refugia Model [43, 44], which was postulated to explain the diversity in the Amazon forest. In the last decade, interest in clarifying the origin of this biodiversity has increased [32]. The refugia hypothesis has been fully proposed and tested [34, 45], and other recent studies on the history of the Neotropical biota in general, searching for both temporal and spatial biogeographic patterns, also propose the uplift of the Andes, rise of land bridges between North and South American marine incursions, and Pleistocene climate changes [46–50] as events of diversification for Neotropical biota. All hypotheses are based on some provisional reductions in gene flow among populations, which promoted divergence in allopatry, leading to different populations because they were somehow geographically isolated [51]. Phylogeographic studies of widespread species in the Brazilian Atlantic Forest occupying more than one phytogeographical region, as *P*. *sylveirae*, may reveal useful information regarding how such species responded to past climatic changes and how these events may have driven the evolutionary history of populations [52].

Given the widespread (east and central regions of Brazil) and the peculiar distribution among Epiponini wasps (absent in the Amazon region), in the present study, we used an integrative approach to reconstruct the evolutionary history of *P*. *sylveirae* in a spatial-temporal framework. The aim of this study was to answer the following questions to investigate the phylogeographic and demographic history of this species: (1) Do populations of *P*. *sylveirae* present genetic structure? (2) If yes, is this structure related to geography? (3) Does the genetic structure also lead to the evolution of distinct morphologic lineages? (4) Is the genetic diversity of this species a result of past habitat fragmentation or recent range expansion? (5) Is there some historical event(s) in the Neotropical region that can be related to the observed phylogeographic pattern?

## Materials and methods

### Sampling and laboratory procedures

Wasps were actively collected along the distributional area of the species with entomological nets in the vegetation or directly from nests and preserved in ethanol (96–100%). Only worker wasps were collected to avoid bias in morphometric analysis due to the dimorphism among castes [26]. One sample was also obtained from a collection of the American Museum of Natural History (Table 1, Fig 2). All the samples were collected with the permission of IBAMA (permit n°10739–1). The collection of specimens in private areas was carried out with the knowledge and permission of the owner. The field study did not involve endangered or protected species.

**Table 1:** Samples of *Protonectarina sylveirae* used for phylogeographic analysis.

Sampling sites	Abbreviation	Latitude (S)	Longitude (W)	Sample size
Bodoquena, MS, BR	BOD	-20.650	-56.733	10
Bonito, MS, BR	BON	-20.967	-56.700	10
Casa Nova, BA, BR	CANO	-9.161	-40.970	10
Catimbaú, PE, BR	CAT	-8.515	-37.349	2
Dourados, MS, BR	DOU	-22.211	-54.915	10
Guarapuava, PR, BR	GUA	-25.397	-51.457	1
Indiaporã, SP, BR	IND	-19.980	-50.289	9
Lavras, MG, BR	LAV	-21.245	-45.000	2
Londrina, PR, BR	LON	-23.330	-51.165	10
Misiones, AR	MIS	-26.400	-54.633	10
Mossoró, RN, BR	MOS	-5.066	-37.400	10
São Carlos, SP, BR	SACA	-21.985	-47.881	10
São Gonçalo do Rio Abaixo, MG, BR	SGRA	-19.883	-43.367	10
São José do Rio Preto, SP, BR	SJRP	-20.820	-49.378	10
Triúnfo, RS, BR	TRI	-29.700	-51.616	10
Viçosa, MG, BR	VIC	-20.753	-42.881	8

Abbreviation: AR, Argentina; BA, Bahia; BR, Brazil; MG, Minas Gerais; MS, Mato Grosso do Sul; PE, Pernambuco; PR, Paraná; RN, Rio Grande do Norte; RS, Rio Grande do Sul; SP, São Paulo.

DNA was extracted from legs, antennae and mesosomal muscles of wasps using DNeasy Blood & Tissue (Qiagen) and Illustra tissue & cells genomic Prep mini spin (GE) kits. The extracted DNA was used to amplify part of the mitochondrial genes *cytochrome oxidase subunit I* (COI) and *16S* and *12S ribosomal RNA*. PCR was performed using a PCR Ready-To-Go Beads (Amersham Biosciences) kit. Primers used to amplify sequences of COI and 16S were developed by Schulmeister *et al*. [53], whereas for the 12S gene, primers were constructed (S1 Table). PCR products were purified with a “GFX PCR DNA and Gel Band Purification” (Amersham Biosciences) kit. Sequencing was performed by the Centro de Recursos Biológicos e Biologia Genômica (CREBIO) using an automatic sequencer ABI3730 XL DNA Analyzer (Applied Biosystems, Foster City, CA). Sequences were inspected and assembled in Geneious Pro 5.6.4 [54] and aligned in Mafft server [55] (http://mafft.cbrc.jp/alignment/server/).

### Genetic diversity

To quantify the variation in DNA sequences and characterize populations, the following parameters were estimated in DnaSP v. 5.101 [56]: number of polymorphic sites (S), nucleotide diversity(π), average number of nucleotide differences (k), total number of mutations (η), number of haplotypes (h) and the haplotype diversity (Hd). To test whether genetic divergence could be explained by isolation by distance, the Mantel test [57] was applied in IBDWS 3.23 [58], with (1000) replications.

### Haplotype relationship and population structure

To determine the relationships among the haplotypes, a network was constructed in PopART (Population Analysis with Reticulate Trees; http://popart.otago.ac.nz) version 1.7 [59] using the median-joining [60] algorithm to describe graphically the relationship between the distribution of haplotypes and the geographic distribution of the populations of *P*. *sylveirae*. An Analysis of Molecular Variance (AMOVA) was performed to test the presence of populational genetic structure. This analysis is based on F-statistics and assumes the hypothesis that the genetic diversity within two populations is not significantly different from that resulting from the joining of the two populations [61, 62]. Three approaches were used: a global analysis, without any *a priori* population group; grouping populations according to the geography; and grouping populations in groups that resulted from the arrangement obtained in the haplotype network. AMOVA was also performed in PopART.

### Demographic events

To detect signs of recent population expansion or bottlenecks in populations of *P*. *sylveirae*, the neutrality tests of Tajima’s *D* [63, 64], Fu’s F*s* [65] and the population size change test R2 [66] were performed using DnaSP v. 5.101 [56]. Significance of tests was obtained based on 1000 coalescent simulations. A value of *D* = 0 implies in neutrality, whereas a significant value of D can indicate population expansion, bottleneck or heterogeneity in mutation rates [67]. A high negative value of *F*_*S*_ indicates population expansion [65], and small values of R2 are expected under a scenario of population expansion [66]. The performance of the R2 test is superior for a small sample size [66, 68].

The distribution of genetic differences between pairs of haplotypes (*Mismatch* distribution) was performed considering the premise of panmictic populations using the program DnaSP v. 5.101 [56]. In general, multimodal distributions are consistent with demographic stability or multiple expansion events; whereas unimodal distributions commonly indicate that the population underwent a recent population and spatial expansion [69]. The Mismatch distribution was performed using all populations together and by geographic distribution and haplotype groups.

### Divergence time

The time since the most recent common ancestor (TMRCA) was estimated under a Bayesian approach using BEAST 1.8.3 [70]. Analyses were conducted using sequences of all mitochondrial genes (12S, 16S and COI) for populations of *P*. *sylveirae* plus sequences of 3 other species of Epiponini (*Agelaia testacea*, *Synoeca surinama* and *Brachygastra augusti*) as out-groups under the GTR nucleotide substitution model, which was identified as the best model for the data in MEGA 7.0.7 [71]. An uncorrelated relaxed clock under a lognormal relaxed distribution was used. The coalescent tree prior used was the constant size, which is most suitable for trees describing the relationships among individuals in the same population/species. The age of the fossil *Agelaia electra* (20.43 to 13.65 mya) [72] was used for calibration under a normal distribution considering a mean of 17.04 ± 0.5mya. Analyses were conducted using default parameters for the MCMC, setting 10 million generations with 10% of the initial runs excluded. The resulting trees were combined using TreeAnnotator v1.8.0, and the consensus tree with the divergence times was visualized in FigTree v1.4.0 [73].

### Phenotypic analysis

**Fig 3:**
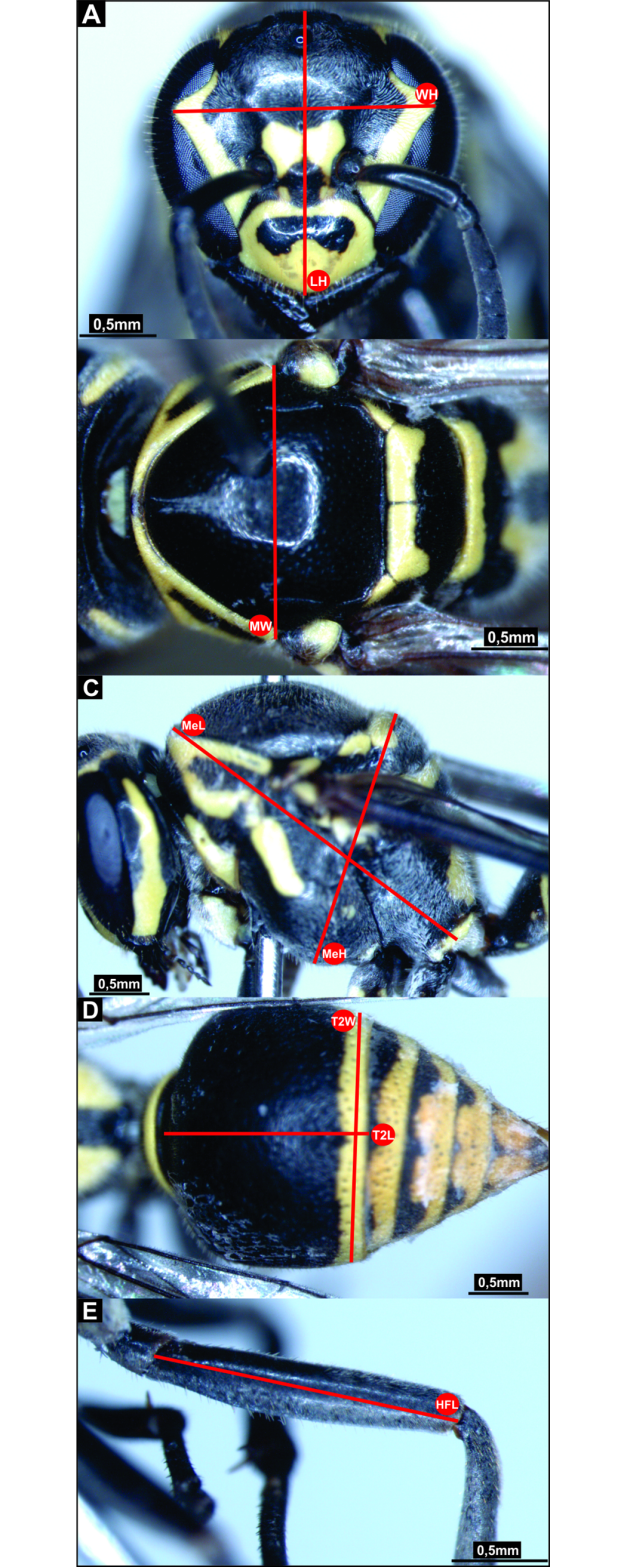
Measurements for morphometry. The red lines represent the body size measures for morphological analysis of populations of *Protonectarina sylveirae*: A. Head length (HL) and head width (HW); B. Mesoscutum width (MW); C. Mesosoma length (MeL) and mesosoma height (MeH); D. 2° metasomal terga length (T2L) and 2° metasomal terga width (T2W); and E. Hind femur length (HFL). Pictures are of a worker from Mossoró, Rio Grande do Norte, Brazil.

Digital images of the bodies of 128 individual *P*. *sylveirae* from the 13 populations sampled (Table 1; populations from Catimbau, Guarapuava and Lavras were not measured because of the few individuals in the samples) were taken witha stereomicroscope coupled with a digital camera (Leica PDF295) before the DNA extraction. The following adult body parts (Fig 3) were measured based on the work of Garcia *et al*. [74]: head length (HL), head width (HW), mesoscutum width (MW); mesosoma length (MeL), mesosoma height (MeH), hind femur length (HFL); 2° metasomal terga length (T2L) and 2° metasomal terga width (T2W). ImageJ software [75] (http://imagej.nih.gov/ij/index.html) was used to derive the measures in the images. Comparisons between populations within the species were performed. To determine the degree of discrimination and identify which measures best explained the data, a multivariate discriminant analysis with a posterior canonical discriminant analysis was performed [76]. The tests were performed with the program STATISTICA 7 [77].

## Results

### Characterization and genetic diversity

The final alignment containing the concatenated data of 12S, 16S and COI resulted in a matrix with 117 sequences and 2320 base pairs. Sequences were submitted to GenBank (S2 Table). A total of 35 haplotypes were identified with a high diversity (0.921 ± 0.015) (Tables 2 and 3). By contrast, nucleotide diversity was low (0.0144 ± 0.00078) across all populations, and the average difference in the number of nucleotides among sequences was 33,496 (Table 3).

**Table 2:** Number of haplotypes for each sample from the concatenated data (12S, 16S and COI genes).

Place	No. of haplotypes	Haplotypes (n)
Bodoquena, MS, BR	1	Hap_6 (10)
Bonito, MS, BR	3	Hap_6 (7), Hap_13 (1), Hap_14 (1)
Casa Nova, BA, BR	4	Hap_16 (1), Hap_17 (1), Hap_18 (1), Hap_19 (1)
Catimbaú, PE, BR	2	Hap_34 (1), Hap_35 (1)
Dourados, MS, BR	2	Hap_2 (9), Hap_3 (1)
Guarapuava, PR, BR	1	Hap_33 (1)
Indiaporã, SP, BR	1	Hap_6 (9)
Lavras, MG, BR	1	Hap_15 (2)
Londrina, PR, BR	2	Hap_22 (8), Hap_23 (2)
Misiones, AR	3	Hap_24 (1), Hap_25 (1), Hap_26 (1)
Mossoró, RN, BR	3	Hap_30 (6), Hap_31 (3), Hap_32 (1)
São Carlos, SP, BR	2	Hap_20(8), Hap_21(2)
São Gonçalo do Rio Abaixo, MG, BR	1	Hap_1(9)
São José do Rio Preto, SP, BR	2	Hap_4(9), Hap_5(1)
Triúnfo, RS, BR	3	Hap_27(5), Hap_28(4), Hap_29(1)
Viçosa, MG, BR	7	Hap_1(1), Hap_7(1), Hap_8(1), Hap_9(2), Hap_10(1), Hap_11(1), Hap_12(1)

Number and designation of haplotypes (Hap) for each sample followed by the number of individuals (n). AR, Argentina; BA, Bahia; BR, Brazil; MG, Minas Gerais; MS, Mato Grosso do Sul; PR, Paraná; RN, Rio Grande do Norte; RS, Rio Grande do Sul; SP, São Paulo.

**Table 3:** Genetic diversity and neutrality tests for total samples and for geographic and haplogroups of *Protonectarina sylveirae* (according to the haplotype network).

		Genetic Diversity					Neutrality Tests*		
Sample	N	H (no. in network)	Hd	Π	S	K	Tajima’s D	Fu’s Fs	R2
**Total**	117	35 (1–35)	0,921±0,015	0,01445±0,00078	144	33,496	-0,08987	-0,70984	0,08917
**A**	24	9 (2, 3, 24–29, 33)	0,812±0,058	0,00417±0,00028	27	9,66304	-0,06530	0,03461	0,12437
**B**	10	2 (22, 23)	0,356±0,159	0,00077±0,00034	5	1,77778	-0,08276	0,21385	0,19208
**C/CE**	67	15 (1, 4–15, 20, 21)	0,802±0,037	0,00430±0,00029	38	9,9638	-0,10060	-0,26695	0,09959
**D**	10	3 (30–32)	0,600±0,131	0,00029±0,00008	2	0,66667	-0,03511	0,24600	0,22369
**E**	4	4 (16–19)	1,000±0,177	0,01661±0,00736	74	38,5000	-0,01890	2,02953	0,24360
**F**	2	2 (34, 35)	1,000±0,500	0,00086±0,00043	2	2,0000	—	0,81599	0,31076
**NE**	16	9 (16–19, 30–32, 34, 35)	0,850±0,077	0,01275±0,00299	103	29,5583	-0,11091	0,17213	0,13588
**SO**	34	11 (2, 3, 22–29, 33)	0,856±0,034	0,00876±0,00066	56	20,3226	-0,03782	-0,24629	0,11423

* Average number.

**Fig 4:**
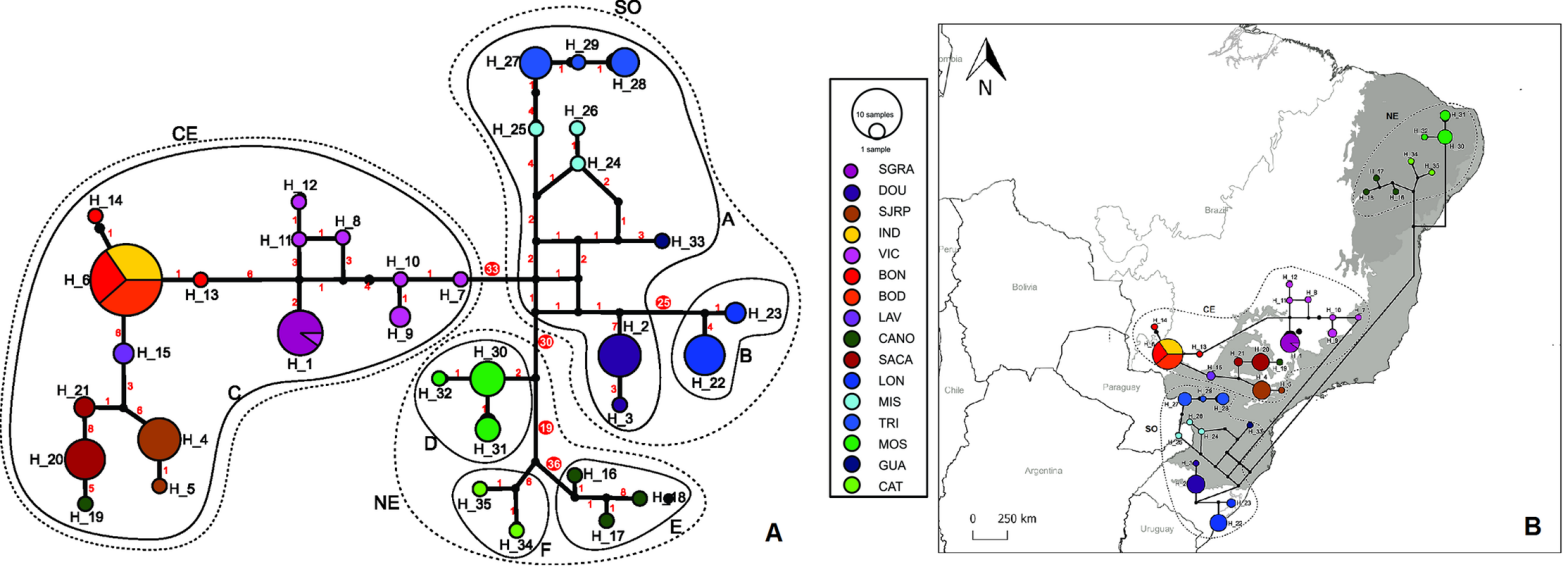
Haplotype relationship. **A.** Haplotype network inferred for haplotypes (H_1 to H_35) of the 12S, 16S and COI genes of *Protonectarina sylveirae*, estimated by the median-joining algorithm. Circles are proportional to the frequency of haplotypes, and colors represent each area sampled. The red numbers on the branches indicate mutational steps between haplotypes. Black circles are median vectors or hypothetical haplotypes (lost or unsampled). Size and positions of some branches were changed for better visualization. The haplotype groups are delimited by full lines, and the geographical ones by dashed lines. Acronyms shown in Table 1. **B.** The same network with the haplotypes superimposed on their approximate geographical location. Areas of Atlantic Forest (dark gray) and Caatinga (light gray) are represented, as well as the geographic groups (dotted lines) of Northeast (NE), Central (CE) and South (SO). The map was generated using the software QGIS v2.18 (http://www.qgis.org/en/site/forusers/download.html).

N: number of samples; H: number of haplotypes; Hd: haplotype diversity; π: nucleotide diversity; S: number of variable sites; k: average number of nucleotide differences; ±: standard deviation. Values of p-value: R2: 0,000; Tajima’s D and Fu’s Fs: not significant. Groups shown in Fig 4.

Samples of BON, BOD and IND shared the same haplotype (Hap 6), which was the most widespread haplotype geographically. Additionally, samples of SGRA and VIC shared haplotype 1 (Table 2). All the other samples presented an exclusive group of haplotypes that were not shared among other samples. The high haplotype diversity and low nucleotide diversity values indicated only small differences between haplotypes; therefore, each population carried a different part of the total amount of diversity of the species.

The Mantel test showed performed for all the samples, no correlation between genetic distance and geographical distance, indicating that the genetic divergence among populations could not be attributed to isolation by distance (S1 Fig).

### Haplotype relationship and populational structure

Fig 4 shows the relationships among haplotypes of *P*. *sylveirae*, with the peculiar haplotype composition for each sample and the presence of “hypothetical haplotypes” (black circles), indicating some haplotypes were already lost, clearly demonstrated. Nevertheless, a lack of samples cannot be discarded.

From the topology of the haplotype network, three groups were delimited according to geography, composed by haplotypes from Northeast (NE), South (SO) and Central (CE) regions of Brazil. Notably, although geographically close to the cities of BON and BOD, haplotypes from DOU were closely related to those from the South (haplogroup C). Some haplotypes were separated by many mutations, including within these geographical groups (e.g., within the NE group). At least six distinct haplogroups, A to G, were delimited (Table 3; Fig 4A). Further analyses were performed based on the network results, considering the division in haplogroups and the grouping by geography (Fig 4B).

Analysis of Molecular Variance (AMOVA) considering all populations as a single group resulted in a high value of F_ST_ (0.948). The source of variation was 94.81% among populations and only 5.19% within populations (Table 4). This result suggested a strong genetic structuration and supported a scenario of low gene flow. When the analysis was repeated grouping the populations according to geography (NE, SW and CE), the F-statistic value was even higher (0.96592), and the percentage of variation was 76% among groups, 20.57% among populations within groups and only 3.41% within populations (Table 4). Analysis performed with samples in the six haplotypes groups based on the topology of the network resulted in an increase in the value of F_CT_ (0.90299) compared with that in the analysis with groups defined by geography (0.76017), andninety percent of the variation was found among the groups (Table 4).

**Table 4:** AMOVA of mtDNA sequences for geographic and haplogroups of *Protonectarina sylveirae*.

Groups	Variation (%)	F-statistic		
		Φ_ST_	Φ_SC_	Φ_CT_
**Total**				
Among population	94.81	0.94811	—	—
Between populations	5.19			
**Geographical groups**				
Among groups	76.02	0.96592	0.85792	0.76017
Among populations within groups	20.57			
Within a population within groups	3.41			
**Haplotype groups**				
Among groups	90.30	0.96645	0.65415	0.90299
Among populations within groups	6.35			
Within a population within groups	3.35			

P-value < 0.001 for all results, based on 1000 permutations. Φ_ST_, variation within populations; Φ_SC_, variation among populations within groups; Φ_CT_, variation among groups.

### Demographic events

**Fig 5:**
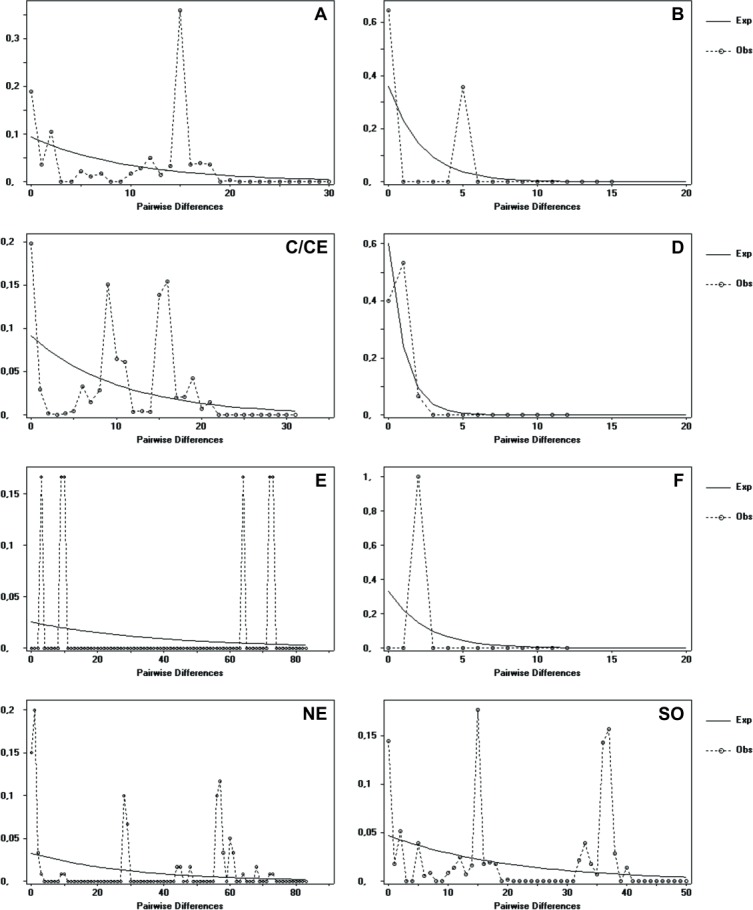
Mismatch distribution. Graphics of pairwise distribution referent to geographic (NE, SO and CE) and haplotype groups (A-F) of *Protonectarina sylveirae*. Observed frequencies of pairwise differences (dotted line) are compared against expected frequencies under a scenario of expanding population sizes (full line).

The *Mismatch* distribution for overall samples resulted in a multimodal distribution, which suggested populational stability. The same pattern was found for the three groups according to geography. A multimodal pattern was also found for haplogroups C and E, in contrast to groups A, B and F that exhibited a unimodal pattern, suggesting a recent demographic expansion [69, 78] or a range expansion with high levels of migration among neighborhoods [79, 80] (Fig 5). Of note, the South region contained A and B groups. For haplogroup D, the observed pattern was similar to that expected.

Values of Tajima’s D and Fu’s Fs were not significant for any of the groups, indicating that the null hypothesis of population neutrality could not be rejected (Table 3). However, the low values of R2 suggested recent expansion (Table 3).

### Divergence time

**Fig 6:**
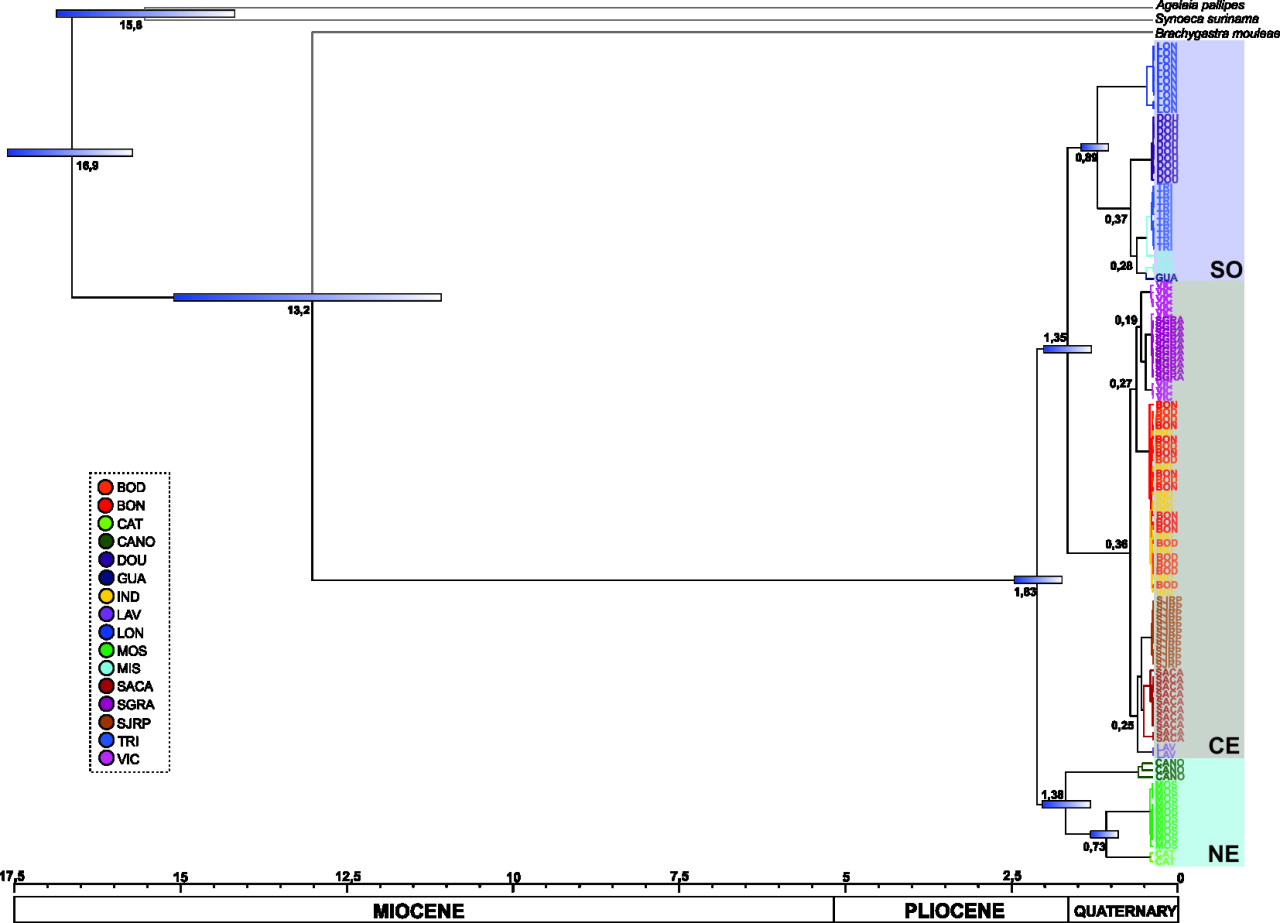
Chronogram with divergence times. Colors of the clades correspond to those of the haplotype network. Numbers annotated on each node represent the divergence times between clades, and the standard deviations (95% of high posterior density) are represented by the blue bars (divergence times less than 100 kaand intervals shorter than 500 ka are not represented). Acronyms shown in Table 1.

The divergence dating analysis revealed a middle Miocene origin for *Protonectarina* at approximately 11 mya. Divergence among the analyzed samples began at approximately 2 mya, with the oldest divergence occurring between lineages of the Northeast (CANO, CAT and MOS) and the others (at 1.83 mya), followed by the separation between lineages from the South (GUA, MIS, TRI, LON and DOU) and the others (at approximately 1.35 mya). More recently, at 0.36 mya, the Central lineages became separated in two primary groups: SACA, SJRP and LAV and BON, BOD, IND, SGRA and VIC (Fig 6).

### Morphological differences

Discriminant analysis showed variation among different populations. Populations of DOU and CANO presented a classification percentage of 100%, which according to the model, indicated these populations were not similar to any other population. By contrast, populations of VIC, LON, MIS and MOS presented a classification percentage lower than 60%. Despite the classification of 37.5%, individuals of the VIC population were morphologically similar to those from IND, BOD and SACA, populations in the same geographical group and haplogroup in which VIC was inserted. Populations of LON and MIS, in the SO group, were classified with a percentage of 30% and 50%, respectively. These populations shared similarities with populations of different groups but were classified more often as more similar to one another. Lastly, the MOS population was similar to populations of BON, BOD, SGRA, VIC, SJRP and SACA (S3 Table).

Contributions of each variable (measure) in functions generated by the value of Wilks’ λ were determined to identify those that were important to discrimination among groups (populations), and only the measures MeL and MeH were not significant (Table 5). Analysis of standardized coefficients identified MW as the most relevant in delimitation of the groups with the highest contribution in the share of discrimination (approximately 80%) represented by two functions discriminating between groups (Root 1 and Root 2) (S4 Table).

**Table 5:** Discriminant function analysis summary.

	MW	T2W	HFL	LH	WH	T2L	MeH	MeL*
**Wilks’ λ**	0,0500	0,0053	0,0056	0,0066	0,0064	0,0045	0,0040	0,0030
**Partial λ**	0,0673	0,6263	0,5958	0,5060	0,5238	0,7342	0,8330	0,9026
**F**	123,46	5,3202	6,0471	8,7023	8,1044	3,2269	1,7864	0,9528
**p-value**	0,0000	0,0000	0,0000	0,0000	0,0000	0,0005	0,0594	0,4982

HL, head length; HW, head width; MW, mesoscutum width; MeL, mesosoma length; MeH, mesosoma height; HFL, hind femur length; T2L, 2° metasomal terga length and T2W, 2°metasomal terga width.Number of variables in model:7,; Grouping: localities (13); Wilks’ λ: ≈0,00337; F (84,663) = 12,106; p <0,0000 is considered significant.

* variable currently not in the model.

**Fig 7:**
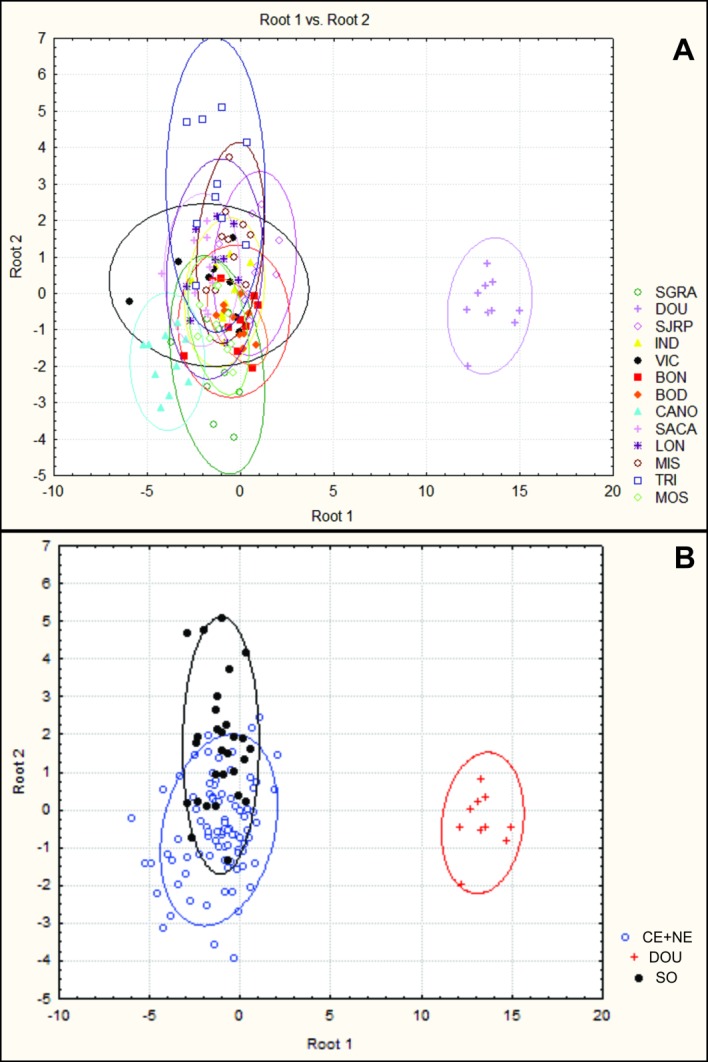
Divergence pattern of groups according to discriminant functions. A. Divergence pattern per sample (represented by the colored shapes). B. Divergence pattern per group showing the upper position of populations of SO (South group, except population of DOU) compared with the other groups. BOD, Bodoquena; BON, Bonito; CANO, Casa Nova; DOU, Dourados; IND, Indiaporã; LON, Londrina; MIS, Misiones; MOS, Mossoró; SACA, São Carlos; SGRA, São Gonçalo do Rio Abaixo; SJRP, São José do Rio Preto; TRI, Triunfo; and VIC, Viçosa.

The ordination plots (Fig 7) show the geographical variation, explained primarily by the shape. A remarkable difference between populations of DOU and those of CANO, although not as conspicuous, can be observed. For the remaining populations, the plots showed no discrete clusters of individuals, with some degree of overlap among them. However, populations of the SO group (LON, MIS and TRI) were distributed from 0 to positive values on root 2 axes, while most populations of CS and NE groups were below 0 (except VIC, SJRP and SACA) (Fig 7B), which indicated that some morphological distinction was evident between the SO group and the other populations.

## Discussion

### Distribution, demography, and genetic and spatial structure

The evolutionary history and the pattern of genetic variability for the species *Protonectarina sylveirae* were clarified in this study. Across the distribution range, populations were genetically highly structured, as confirmed by AMOVA, presumably reflecting long lasting isolation in the past. Populations within the same geographical region were genetically more similar, such as populations of BON and BOD, or presented a low degree of genetic divergence, suggesting restricted gene flow between geographically separated groups of populations. However, the haplotype composition for most populations inside the same geographical region implied a condition of restricted gene flow not related to isolation by distance but most likely caused by a barrier. A unique haplotype composition was found for populations of *P*. *sylveirae*, in which exclusive alleles were found across most of the populations. A similar pattern was found for *Eugenia uniflora* from the south of the Atlantic Forest, which became geographicaly isolated during glacial cycles in fragments with appropriate environmental conditions, resulting in lineage divergence [5]. In the same way, the pattern found for *P*. *sylveirae* suggests a long-term persistence of these populations, rather than recent migrations. Moreover, the founding-nest behavior of reproductive females most likely reinforced the highly structured pattern of *P*. *sylveirae*. Indeed, individuals sampled in geographically close nests shared very similar haplotypes with low nucleotide diversity, which is also found within some species of ants with restricted female dispersion [81–84].

A swarm has a limited dispersion capacity once the queen is constrained by the dispersing ability of nestmates [85]. The low dispersal capacity of the queen can result in population genetic viscosity (in a continuous population, the closest colonies present greater genetic similarity than the colonies that are more distant from each other) and inbreeding depression (a decrease in the fitness of inbred individuals) [22, 86]. The latter condition is especially important in social insects because colonies present small effective population sizes [87]. However, because males can be efficient dispersers (they leave the home nest to find foreign colonies), the effects of low dispersion of queens, genetic viscosity, and inbreeding can be mitigated. This can be observed, for example, in biparentally inherited (nuclear) genes, which do not present genetic viscosity [85].

Lineages of *P*. *sylveirae* showed low haplotype sharing and long-term divergence. This divergence is not surprising when the distribution and the molecular markers of this species are considered. The use of only mitochondrial markers has some limitations, mainly because it represents the study of a single locus, showing only a small part of the history of populations and reflecting the matrilineal history, which may differ from the population's history when analyzing, for example, nuclear genes [88, 89]. Moreover, because the effective population size of mtDNA is a fourth of that of nuclear autosomal sequences, the process of lineage sorting (when a species splits, allelic lineages sort into the descendant populations) occurs much faster and the rate of allele loss is high for mtDNA lineages [88].

Although reciprocal monophyly of the alleles at a given locus in allopatrically or parapatrically distributed sets of populations is commonly interpreted as an indication of distinct species [90, 91], other conditions can lead to this pattern, as in the case of mitochondrial DNA. When the locus is maternally inherited, strong genetic structure can also result from low dispersal distances of females even when autosomal and paternally inherited genes are regularly exchanged between the same sets of populations [92]. Thereby, two or more species might be inferred although the populations in question form a single metapopulation lineage [93]. Decreasing population size also increases the chance of observing phylogeographic breaks. Maternally inherited markers have one-fourth the effective population size of nuclear genes and therefore are more likely to show phylogeographic structure in a continuously distributed species [92]. For social insects, the effective population size is even smaller, because among all the members of the colony, only a small percentage reproduces.

The type of social organization in vespids has generated profound effects in several aspects of the group [74]. Non-swarm-founding species can generate new colonies rapidly and their dispersion could be facilitated; however, the colony success is low given the low number of founding individuals [19]. By contrast, a swarm-founding species, such as *P*. *sylveirae*, reduces queen mortality risk and increases homeostatic control of the colony, but the dispersal process could be restricted with populations prone to local extinction because the swarm is vulnerable to harsh climatic conditions during the migration [19, 94]. Nests of *P*. *sylveirae* are not common and are not easily located in the field, with nests usually built in high trees and found only in areas with forested vegetation. Even samples collected in dry environments, such as those from CANO, CAT and MOS in the Caatinga biome, were found in high trees. These biological requirements likely shaped the distribution of populations and contributed to the low rates of gene flow among populations. Areas with open vegetation and/or dry conditions most likely were not a barrier for the expansion of populations but were for the establishment of colonies.

Sufficient gene flow between populations can slow down or prevent the process of geographic differentiation and leave a signature of little population structure over large areas. This signature is commonly observed in flying insect species, specifically in those species that migrate or are good dispersers [95]. Nevertheless, this pattern was not observed for *P*. *sylveirae*. The divergence among haplotypes and the genetic structuration suggested a deep divergence instead of ancestry based on coalescence, indicating low rates of gene flow and resulting in the high index of F_ST_. Considering the predictions of the theory of coalescence, a direct relationship occurs between haplotype frequency and the age of haplotypes in which the most common haplotypes are the oldest [96]. Hence, most of new mutants are derivatives of common haplotypes; therefore, rare variants are the most recent mutations and most closely related to the common haplotypes than to the other rare variants [97]. In this study, the haplotype ancestry of groups of haplotypes could not be determined. The presence of hypothetical haplotypes (median vectors) can be biologically interpreted as possibly extant unsampled sequences or extinct ancestral sequences [60]. Nevertheless, a lack of samples cannot be discarded, primarily in the area close to the southeast coast, which was not sampled.

Rapid cycles of extinction and colonization can also affect the genetic structure of populations in which frequent founding events during colonization may increase the value of F_ST_ [98]. Nevertheless, this value depends on the settlement mode, more specifically the source of the origin of migrants [99]. The combination of high haplotype diversity (Hd) and low nucleotide diversity (π), as observed in our data, also can be a signature of a rapid demographic expansion from a small effective population size [100]. In eusocial species, such as *P*. *sylveirae*, only a few individuals of the colonies are reproductive. After a swarm, colonies start with several queens and workers, and as the colony develops, some queens disappear or adopt a worker role, and the number of queens reduces until one or a few queens remain [17]. Carvalho [101] also found a high value of Hd and a low π for *Angiopolybia pallens* and *Synoeca surinama*, two species of paper wasps in the same tribe as *Protonectarina*. The author found a lower genetic diversity for *S*. *surinama* than that for *A*. *pallens*, which was attributed to the differences in biology between the two species (e.g., vagility, body size and distinct colonization systems). In the same way, both genera diverge earlier than *Protonectarina* [102] and show differences in many aspects, such as in nest construction, number of individuals in the colony and behavior.

The multimodal Mismatch distribution suggested stability in populations [69, 79]. However, recent changes in the size of a population cannot be detected because of threshold effects, lapses of time or previous demographic events, which can mask the effects of recent events [103]. Multimodal distribution can also be generated for populations that experienced recent population expansion but are subject to higher migration rates and/or suffered historical population reduction [104–105].

Morphometric analysis indicated differences among populations, which were statistically significant, although morphologically imperceptible. Furthermore, according to the results, the selected measures were adequate for discrimination of groups. From the graphic of discriminant analysis (Fig 7), a difference in the distribution of plots between the SO group (except DOU) and the other populations could be observed. The size and shape of the DOU sample were highly divergent from those of the other populations. Phylogeographic structure with little or absence of phenotypic variation can occur when stabilizing selection is acting, which tends to keep the population constant over time, increasing the fitness of the members with average characteristics (more common) within the population compared with those with extreme characteristics [10, 106]. When strong stabilizing selection acts on traits that characterize the niche of a species, populations will track suitable habitat as it appears and disappears over time [107]. Furthermore, the presence of strong biogeographic barriers to gene flow between stable environments, which exhibit little variation in space and time, allow species to be subdivided into populations and accumulate genetic differences without any morphological differentiation [107]. This condition can generate “cryptic” lineages and later, species [10, 108].

Considering other morphological traits, such as cuticular punctuation and color, no different patterns were observed among populations. In fact, color showed intra-population differences. Several studies reveal deep intraspecific phylogeographical structure with multiple lineages (phylogroups) [52], which can be indicative of cryptic species, i.e., distinct species that are morphologically indistinguishable [108]. However, we believe that this type of affirmation requires a more accurate study.

Differences concerning patterns obtained from molecular and morphological data rely on the way in which selection acts to drive evolution [109]. Functional genes can be under strong stabilizing selection, decreasing the rate of evolution [110, 111], whereas the non-functional regions can rapidly evolve according to the neutral evolution model. By contrast, phenotypic evolution is mostly driven by natural selection, which can force morphological evolution away from the expected correlation with molecular data [109, 112]. The possible outcomes range from cryptic lineages resulting from stabilizing selection [113] to high rates of morphological divergence because of directional selection [114].

### Time, diversification and historical aspects

The dating analysis showed a time of origin of the species in the Middle Miocene (approximately 13 mya). During the Miocene, one of the largest and most extensive humid environments in geological history was formed in the Amazon region. The evolution of this system was influenced primarily by the rise of the Andes in association with other factors such as global sea level change, subsidence and a seasonally humid climate [115]. In the Middle/Late Miocene (14–10 mya), in a relatively warm and humid phase of Earth [116], the system connected with the Atlantic Ocean, covering extensive areas between the Andean Cordillera and the Guiana and Brazilian shields [115, 117]. In the periphery of these areas, lands emerged in which tropical forest prevailed [115]. This event most likely isolated the ancestor of *P*. *sylveirae* in the eastern part of Brazil, explaining the absence in the Amazon region.

Divergence times estimated for the primary genetic breaks were placed within the Plio–Pleistocene periods (approximately 2 mya), a period in which the average temperature of the earth began to decrease rapidly and for which extensive records document changes in forest distribution associated with climatic cycles [45, 116, 118–126]. The first break occurred between NE and SO+CE groups, with the northeast lineages of *P*. *sylveirae* the oldest ones. Analysis of palynological records for areas of the Caatinga in the states of Bahia and Pernambuco showed hot and wet weather [127–129] until approximately 4000 years ago, when the modern Caatinga expanded. Moreover, in the Pliocene/Holocene boundary, a dense arboreal vegetation of the ombrophilous forest type predominated under a humid tropical climate [127]. For Catimbau National Park in Pernambuco, the humid condition continued until approximately 1000 years ago, and a constant humidity persists today [128, 129]. These environmental conditions were certainly suitable for the occurrence of *P*. *sylveirae* populations.

From the current vegetation, the Caatinga is clearly inhospitable to most rainforest species and even more stressful to arboreal ones [130]. Living in areas of arboreal Caatinga, populations in this biome undergo sudden changes in the environment throughout the year. In lineages in other locations in which wasps are confined to areas that are more humid, such adaptability does not occur. A notable characteristic of this species, related to survival in periods of scarce resources, is the production and storage of a large amount of honey, unusual for paper wasps [16]. Climatic changes during Quaternary period, which caused isolation of forested areas, most likely contributed to the increase in genetic divergence among populations of this region. A vast fossil record of mammals provides strong evidence of a past (Pleistocene) mosaic of vegetation of open fields, savannas and forests in the Caatinga [128, 131]. Furthermore, the called “Brejos de Altitude” montane forests that currently exist within the semi-arid region are relicts of an ancient and wider forest cover and were forest refuges during the Plio-Pleistocene [42, 132].

Climatic fluctuations and the effect of Pleistocene arid phases were greater in the southern portion of the biome than in the northern region [34, 133], which acted as a refuge because of the environmental stability [34]. Thus, populations from the south should present less genetic diversity than populations from north and central regions [34], which was not observed for *P*. *sylveirae*. The Atlantic Forest may have been able to maintain a reasonable degree of continuity during such climatic events because of the complex topography of the region and the potential for vertical migration [133]. The geographic distribution for most lineages of *P*. *sylveirae* included altitudes from 400 to more than 1000 m, which can be explained by vertical migration. Based on the mountain refuge hypothesis [134, 135], during dry periods, forest formations were more likely to occur in high altitude areas because of increased rainfall resulting from the orographic effect [136]. Moreover, the Last Glacial Maximum (~20,000Ka) is a period too recent to explain the major genetic breaks that occurred among lineages of *P*. *sylveirae*.

The DOU population was in the SO group, and these populations share the Paraná watershed and a common climate region. However, the DOU population was morphologically different from the other populations, including those of the same group. A possible cause is the Paraná River between DOU and the other SW populations, which could have hindered gene flow, reinforcing the differences among populations. Another notable result was that, despite the spatial proximity, the population of DOU was not in the same haplogroup of populations of BON and BOD. However, BON and BOD are in a different watershed (basin of Paraguay). Moreover, DOU is in the plateau zone of the state of Mato Grosso do Sul, whereas BON and BOD are in Serra da Bodoquena, which are two different geological formations.

Previous studies with other genera of Epiponini also found a recent time of divergence. A phylogeographic analysis of *Angiopolybia pallens* and *Synoeca surinama* [101] found early origins for the species, approximately 3 and 2 mya, respectively. Menezes *et al*. [137] found a middle/late Miocene origin for *Synoeca*, with subsequent diversification of extant species occurring in the Plio-Pleistocene. The calibration used was different for both works and also differed in this study. The inference of lineage divergence time from molecular data has been criticized, primarily because dating is prone to many types of error (particularly for those taxa lacking an available fossil record for calibration) [52], one of which is related to the high influence of the calibration scheme [138]. Hence, the patterns reported here must be evaluated carefully.

## Conclusions

The genetic diversity of *Protonectarina sylveirae* presented a strong structuration, suggesting the presence of barriers, which restricted or even interrupted the genetic flow among populations. Moreover, deep phylogeographical divisions within taxa also suggested extensive periods of isolation among populations of the species. The possibility of genetic divergence because of isolation by distance was rejected. From the haplotype network, three primary groups were delimited based on geography: Northeast, South and Central. However, at least six haplogroups were delimited genetically. Demographic analysis showed stability for most populations, indicating that the populations had remained constant throughout their evolutionary history. However, signs of recent expansion were found for populations of the south region and Catimbaú. Importantly, note that the eusociality of *P*. *sylveirae* also strongly influenced genetic and geographic patterns. Despite the uniformity in phenotypes, a small degree of morphological differentiation was also found for populations in morphometric analysis, revealing a distinction between populations of SO and the others and a particular morphology for the sample of DOU. No differences in other morphological features such as color or punctuation were observed for different patterns among populations.

The dating analysis indicated the origin of *Protonectarina* in the limit of Middle/Late Miocene, approximately 11 mya. In this period, an extensive marine ingression acted as a barrier, which could explain the absence of the species in the Amazon region. Divergence of haplogroups began from the Plio/Pleistocene boundary. No clear overall pattern indicated the origin of most of the South American species during the Pleistocene, as predicted by the refuge theory. However, the last glacial maximum (LGM) most likely modeled the current distribution of species, as observed for *P*. *sylveirae*. Nevertheless, the climatic changes that occurred in the LGM could not be the cause of genetic breaks among lineages because that event occurred more recently than the divergence found in our analysis. The divergence times obtained might provide a crude estimate of the relative order of events isolating the major haplotype lineages of *P*. *sylveirae* in the Brazilian Atlantic Forest.

Our results provide insights on the phylogeography of *Protonectarina sylveirae* and increase understanding of the complex pattern of diversification for the enormous biodiversity in the Brazilian Atlantic Forest. Further studies using a multiple loci approach and increasing the range of geographical sampling of the species will provide a more complete picture of the evolution and diversification of paper wasps and contribute more pieces to solve the jigsaw of historical diversification of biota in South America.

## Supporting information

S1 Fig Graphics of Mantel test to identify isolation by distance. Lines represents RMA regression. The values of p were not significant for any of the analyzes. Z, Pearson's correlation coefficient between matrix; r, Pearson’s statistics.
(TIF)

S1 Table Sequence of primers and used PCR conditions for amplify fragments of genes. F, *forward*; R, *reverse*; bp, base pair; *approximate size of the amplicon. COI, cytochrome oxidase subunit I; 12S ribosomal RNA, 16S ribosomal RNA.
(DOCX)

S2 Table GenBank accession numbers. Accession numbers provided after submission of obtained gene sequences.
(DOCX)

S3 Table Classification matrix according the discriminant model. The matrix shows, for each population, the number of cases classified correctly and incorrectly according the discriminant model. Rows: observed classification; columns: predicted classification.
(DOCX)

S4 Table Standardized coefficients for the canonical variables. Numbers in bold show the contribution of the two functions that best explained the discrimination found among populations. HL, head length; HW, head width; MW, mesoscutum width; MeL, mesosoma; MeH, mesossoma height; HFL, hind femur lenght; T2L, 2° metasomal terga length and T2W, 2° metasomal terga width.
(DOCX)
