# Supplementary material for: Phylogeographic analysis reveals high genetic structure with uniform phenotypes in the paper wasp Protonectarina sylveirae (Hymenoptera: Vespidae)
Source: PLoS One. 2018 Mar 14;13(3):e0194424. doi: 10.1371/journal.pone.0194424 (PMC5851647; doi:10.1371/journal.pone.0194424)
Supplement: S1 Table — F, forward; R, reverse; bp, base pair; *approximate size of the amplicon. COI, cytochrome oxidase subunit I; 12S ribosomal RNA, 16S ribosomal RNA. (DOCX) [file pone.0194424.s002.docx]

| **Gene** | **Primer** | | | | **Step 1** | **Step 2** | | | | **Step 3** |
| --- | --- | --- | --- | --- | --- | --- | --- | --- | --- | --- |
|  | **direc.** | **name** | **Sequence (5’-3’)** | **Lenght**  **(pb)** | **Initial denaturation** | **Denaturation** | **Annealing** | **Extention** | **N° of cicles** | **Final extention** |
| 12S | F  R | 12SF  12SR | ACTWTGTTACGACTTATTYC  AAACTAGGATTAGATACCCTATTAT | ~400 | 94˚C/2 min. | 94˚C/30s. | 49˚C/15s. | 68˚C/30s. | 35 | 72˚C/7 min. |
| 16S | F  R | 16SA  16SB | CGCCTGTTTATCAAAAACAT  CTCCGGTTTGAACTAAGATCA | ~515 | 94˚C/2 min. | 94˚C/15s. | 47˚C/15s. | 72˚C/15s. | 40 | 72˚C/6 min. |
| COI (f1) | F  R | JERRY  PAT | CAACATTTATTTTGATTTTTTGG  TCCAATGCACTAATCTGCCATATTA | ~850 | 94˚C/2 min. | 94˚C/30s. | 48˚C/30s. | 72˚C/45s. | 40 | 72˚C/7 min. |
| COI (f2) | F  R | LCO  HCO | GGTCAACAAATCATAAAGATATTGG  GTAAATATATGRTGDGCTC | ~740 | 94˚C/5 min. | 94˚C/45s. | 51˚C/45s. | 72˚C/1,5min. | 35 |  |
