# Supplementary material for: Phylogeographic analysis reveals high genetic structure with uniform phenotypes in the paper wasp Protonectarina sylveirae (Hymenoptera: Vespidae)
Source: PLoS One. 2018 Mar 14;13(3):e0194424. doi: 10.1371/journal.pone.0194424 (PMC5851647; doi:10.1371/journal.pone.0194424)
Supplement: S2 Table — Accession numbers provided after submission of obtained gene sequences. (DOCX) [file pone.0194424.s003.docx]

| **Sequence** | **Accession number** |
| --- | --- |
| 12S population | MF134898 – MF135014 |
| 12S outgroup | MF987814 – MF987816 |
| 16S population | MF135015 – MF135130 |
| 16S outgroup | MF987817 – MF987819 |
| COI population | MG432243 – MG432358 |
| COI_outgroup | MG456607 – MG456609 |
