# Supplementary material for: Phylogeographic analysis reveals high genetic structure with uniform phenotypes in the paper wasp Protonectarina sylveirae (Hymenoptera: Vespidae)
Source: PLoS One. 2018 Mar 14;13(3):e0194424. doi: 10.1371/journal.pone.0194424 (PMC5851647; doi:10.1371/journal.pone.0194424)
Supplement: S3 Table — The matrix shows, for each population, the number of cases classified correctly and incorrectly according the discriminant model. Rows: observed classification; columns: predicted classification. (DOCX) [file pone.0194424.s004.docx]

|  |  | **% correct** | **1** | **2** | **3** | **4** | **5** | **6** | **7** | **8** | **9** | **10** | **11** | **12** | **13** |
| --- | --- | --- | --- | --- | --- | --- | --- | --- | --- | --- | --- | --- | --- | --- | --- |
| **1** | **SGRA** | 80 | 8 | 0 | 0 | 1 | 0 | 0 | 1 | 0 | 0 | 0 | 0 | 0 | 0 |
| **2** | **DOU** | 100 | 0 | 10 | 9 | 0 | 0 | 0 | 0 | 0 | 0 | 0 | 0 | 0 | 0 |
| **3** | **SJRP** | 90 | 0 | 0 | 0 | 0 | 0 | 0 | 0 | 0 | 1 | 0 | 0 | 0 | 0 |
| **4** | **IND** | 70 | 0 | 0 | 0 | 7 | 0 | 0 | 0 | 0 | 0 | 2 | 1 | 0 | 0 |
| **5** | **VIC** | 37,5 | 0 | 0 | 0 | 1 | 3 | 0 | 2 | 0 | 1 | 0 | 0 | 0 | 0 |
| **6** | **BON** | 60 | 0 | 0 | 0 | 0 | 0 | 6 | 1 | 0 | 0 | 0 | 0 | 0 | 2 |
| **7** | **BOD** | 70 | 0 | 0 | 0 | 0 | 0 | 3 | 7 | 0 | 0 | 0 | 0 | 0 | 0 |
| **8** | **CANO** | 100 | 0 | 0 | 0 | 0 | 0 | 0 | 0 | 10 | 0 | 0 | 0 | 0 | 0 |
| **9** | **SACA** | 60 | 0 | 0 | 0 | 0 | 0 | 1 | 0 | 0 | 6 | 2 | 0 | 0 | 1 |
| **10** | **LON** | 30 | 2 | 0 | 0 | 0 | 0 | 0 | 0 | 0 | 1 | 3 | 3 | 0 | 0 |
| **11** | **MIS** | 50 | 0 | 0 | 1 | 0 | 0 | 1 | 0 | 0 | 0 | 3 | 5 | 0 | 0 |
| **12** | **TRI** | 70 | 0 | 0 | 1 | 0 | 0 | 0 | 0 | 0 | 0 | 2 | 0 | 7 | 0 |
| **13** | **MOS** | 50 | 1 | 0 | 1 | 0 | 1 | 1 | 1 | 0 | 1 | 0 | 0 | 0 | 5 |
| **TOTAL** | | 67,187 | 11 | 10 | 14 | 9 | 4 | 12 | 12 | 10 | 10 | 12 | 9 | 7 | 8 |
