# Supplementary material for: Phylogeographic analysis reveals high genetic structure with uniform phenotypes in the paper wasp Protonectarina sylveirae (Hymenoptera: Vespidae)
Source: PLoS One. 2018 Mar 14;13(3):e0194424. doi: 10.1371/journal.pone.0194424 (PMC5851647; doi:10.1371/journal.pone.0194424)
Supplement: S4 Table — Numbers in bold show the contribution of the two functions that best explained the discrimination found among populations. HL, head length; HW, head width; MW, mesoscutum width; MeL, mesosoma; MeH, mesossoma height; HFL, hind femur lenght; T2L, 2° metasomal terga length and T2W, 2° metasomal terga width. (DOCX) [file pone.0194424.s005.docx]

|  | **Root 1** | **Root 2** | **Root 3** | **Root 4** | **Root 5** | **Root 6** | **Root 7** |
| --- | --- | --- | --- | --- | --- | --- | --- |
| **MW** | -1,1656 | 0,0884 | -0,1728 | 0,0884 | 0,0289 | -0,0198 | 0,0491 |
| **LT2** | 0,3227 | -0,5066 | 0,3622 | 0,5529 | -0,5484 | 0,1271 | 0,5188 |
| **HFL** | -0,3360 | 0,6828 | 0,4123 | -1,0566 | -0,2312 | -0,1554 | 0,4351 |
| **LH** | 0,0499 | -1,5386 | -0,3723 | -0,5556 | 0,7282 | -0,6956 | 0,3374 |
| **WH** | 0,2538 | 1,3297 | 0,4361 | 1,1636 | -0,1126 | -0,3059 | -0,4512 |
| **T2L** | 0,1760 | -0,1121 | 0,2878 | 0,3225 | 0,7893 | 0,6920 | 0,0973 |
| **MeH** | 0,1855 | -0,3224 | 0,3261 | -0,1857 | -0,4052 | 0,2809 | -1,0152 |
| **Eigenval** | 18,5192 | 1,9152 | 1,1698 | 0,4240 | 0,3372 | 0,2184 | 0,0352 |
| **Cum. prop.** | 0,8187 | 0,9034 | 0,9551 | 0,9738 | 0,9887 | 0,9984 | 1,0000 |
